# Nocturnality constrains morphological and functional diversity in the eyes of reef fishes

**DOI:** 10.1186/1471-2148-11-338

**Published:** 2011-11-19

**Authors:** Lars Schmitz, Peter C Wainwright

**Affiliations:** 1 Department of Evolution and Ecology, University of California, Davis, CA, 95616, USA; 2 Center for Population Biology, University of California, Davis, CA, 95616, USA

## Abstract

**Background:**

Ambient light levels are often considered to drive the evolution of eye form and function. Diel activity pattern is the main mechanism controlling the visual environment of teleost reef fish, with day-active (diurnal) fish active in well-illuminated conditions, whereas night-active (nocturnal) fish cope with dim light. Physiological optics predicts several specific evolutionary responses to dim-light vision that should be reflected in visual performance features of the eye.

**Results:**

We analyzed a large comparative dataset on morphological traits of the eyes in 265 species of teleost reef fish in 43 different families. The eye morphology of nocturnal reef teleosts is characterized by a syndrome that indicates better light sensitivity, including large relative eye size, high optical ratio and large, rounded pupils. Improved dim-light image formation comes at the cost of reduced depth of focus and reduction of potential accommodative lens movement. Diurnal teleost reef fish, released from the stringent functional requirements of dim-light vision have much higher morphological and optical diversity than nocturnal species, with large ranges of optical ratio, depth of focus, and lens accommodation.

**Conclusions:**

Physical characteristics of the environment are an important factor in the evolution and diversification of the vertebrate eye. Both teleost reef fish and terrestrial amniotes meet the functional requirements of dim-light vision with a similar evolutionary response of morphological and optical modifications. The trade-off between improved dim-light vision and reduced optical diversity may be a key factor in explaining the lower trophic diversity of nocturnal reef teleosts.

## Background

Temporal resource and habitat partitioning is a major axis of ecological diversification in reef fishes [1,2]. The large majority of reef fish families are primarily day-active (diurnal), yet night-active behaviour (nocturnality) has been observed in at least 13 families across teleost reef fishes including such well-known groups as soldier- and squirrelfish (Holocentridae), cardinalfish (Apogonidae), grunts (Haemulidae), and moray eels (Muraenidae) [1,3-6]. Nocturnality comes with the enormous optical challenge of maintaining adequate visual performance at low light levels that severely limit image resolution and brightness [7-9] and compromise image quality. As most reef teleosts are visual foragers [10,11], prey detection becomes increasingly challenging with a reduction of ambient light. Nocturnal reef fishes feature reduced trophic diversity compared to diurnal species, yet despite low light levels nocturnal reef fishes successfully target and capture a variety of different prey items, including large elusive prey, mobile benthic invertebrates, and large zooplankton [2,12-15].

How do nocturnal fish successfully cope with the physical challenge of scotopic (dim-light) vision? The maintenance of good image quality in scotopic vision requires modifications of the visual system [7-9,16] and these requirements are thought to be met in reef fishes by an evolutionary syndrome of several morphological and physiological traits. For example, large eye diameter [17-19], rod-dominated retinae [17,20], and a high degree of convergence of rod photoreceptors on ganglion cells [21] are recognized adaptations to scotopic vision in shallow-water teleosts. Information on eye shape of shallow-water teleosts is sparse in the literature [17,22], in spite of the potential for adaptive modifications at this level of design, as suggested by the extensive research efforts undertaken on the ecomorphology of the chambered eyes of terrestrial amniotes in the last decade [23-27].

Diel activity pattern has been found to strongly influence the morphological evolution of the eyes of terrestrial vertebrates [23,25,27,28]. Even though there are differences in marine and terrestrial vision [16,29], physiological optics predicts that diel activity patterns trigger evolutionary responses resulting in different morphologies of diurnal and nocturnal eyes.

The three main issues that we address in this paper are all related to whether physical characteristics, i.e., contrasting light levels of day and night, are correlated with features of eye morphology in teleost reef fishes. First, we examined whether nocturnal species have larger eyes relative to body mass than diurnal species. Then we tested predictions from optics for how eye morphology could be modified for better performance in dim-light. Third, we tested the prediction that physical and functional requirements of vision in dim light result in reduced morphological diversity of eyes compared to diurnal taxa. In contrast, we predicted that diurnal reef fish have high morphological and optical diversity, because they are released from the physical limitations of low light levels and selection towards this adaptive peak should be weaker. In addition diurnal reef fish may take advantage of adaptive peaks that benefit other aspects of visual performance than light sensitivity. These adaptive peaks may be located in different areas of eye morphospace, which will increase the morphological and functional diversity of diurnal reef teleosts.

## Methods

### Classification of diel activity patterns

We distinguished between two main diel activity patterns: nocturnal (night-active) and diurnal (day-active). We classified all fish that are mainly active at night as nocturnal, and all fish with main activity during the day as diurnal, on the basis of literature surveys [1,3-6,14,30-33] and personal observations (P.C. Wainwright). A finer distinction of diel activity patterns is currently impossible because of the lack of more quantitative behavioural data for most reef fishes (although see [14] and [34] for examples). There is some indication that some fish species, in particular within Serranidae, Scorpaenidae, and Haemulidae, are active both day and night (cathemeral), or twilight-active (crepuscular). As the evidence for this is often anecdotal we refrain from a formal classification for the purpose of this analysis until more data are available.

### Specimens, measurements, and procedures

We sampled 265 species of teleost reef fish in 43 families with a total number of 849 specimens (1-30 individuals per species) for eye morphometrics (Additional file 1). Most species in our sample are mainly reef inhabitants and live in clear marine environments, but a few species also enter murkier brackish and muddy coastal waters, e.g., the silverside *Atherinomorus stipes*, the mojarra *Gerres cinereus*, the haemulids *Plectorhinchus chaetodonoides *and *Orthopristis chrysoptera*, the kyphosid *Microcanthus strigatus*, and the mullet *Mugil cephalus*. The size range across individuals was 44-638 mm standard length. We sampled adults whenever possible, but some specimens were relatively small juveniles. There were 211 diurnal species in the dataset, and 54 nocturnal species. The nocturnal species are from the following 12 families: Apogonidae (9 species), Congridae (1), Diodontidae (1), Haemulidae (13), Holocentridae (10), Lutjanidae (5), Muraenidae (7), Ophichthidae (2), Pempheridae (1), Priacanthidae (2), Sciaenidae (1), Serranidae (2).

**Figure 1:**
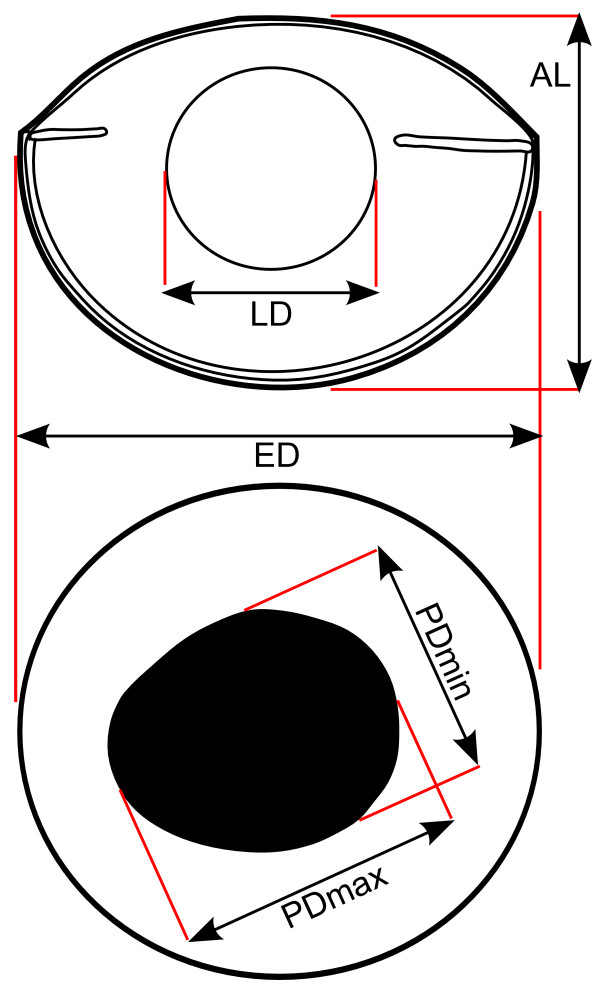
**Illustration of eye measurements**. Schematic illustrations of a teleost eyeball identifying morphological measurements in horizontal cross section (top) and lateral view (bottom). AL axial length, ED eyeball diameter, LD horizontal lens diameter, PDmax largest pupil diameter, PDmin smallest pupil diameter. Modified from [22].

We dissected all specimens shortly after euthanizing them with an overdose of MS-222. We excised the left eyeball first, removed attached ocular muscles, and cut the optic nerve close to the sclera. We measured eyeball diameter, axial length, the largest and smallest pupil diameter, and lens diameter (Figure 1) with an optical micrometer on a Wild binocular stereomicroscope. It should be noted here that the pupil of teleosts is generally considered static, i.e., there is no pupillary response to changes in ambient light, with a few notable exceptions [35]. Then, we cut away iris and cornea, removed the lens from the eye, and measured the equatorial diameter of the lens, again using the optical micrometer. We repeated this procedure for the right eye. All research was carried out in accordance with the UC Davis animal use and care protocols.

### Physiological optics

Optics provides models for light sensitivity on the basis of morphological and physiological features of the eye. Schmitz and Motani [27] introduced the optical ratio (OPT) as a descriptor of light sensitivity, on the basis of earlier work by Hughes and Land [36,37]. OPT is the product of the ratio between the optical aperture (A) and the posterior nodal distance (PND), i.e., the inverse of f-number, and the ratio between optical aperture and the diameter of the retina (RD):

$${\text{OPT = A}}^{\wedge }2∕\text{(RD}\times \text{PND)}.$$

OPT is a useful discriminator between the three main types of ocular image formation in terrestrial amniotes: photopic (image formation in bright light), mesopic (intermediate light), and scotopic (dim light). The form-function relation of OPT and ocular image formation has been tested empirically and found valid by approximating the optical variables with morphological features [27,28,38].

Absolute eye size may also influence light sensitivity under certain conditions. As both OPT and f-number are ratios, they are independent of size unless there are deviations from the optically expected isometry. Nevertheless, a bigger eye may still have better light sensitivity to extended light sources because of a higher degree of neural summation by pooling of photoreceptor signals. The negative effect of signal-pooling on visual acuity could be offset by the increase of the focal length of the eye. Bigger eyes may also have better sensitivity to point light sources such as bioluminescent flashes, because of their absolutely larger apertures. In contrast to sensitivity to extended light sources, point light detection is independent from retinal area and focal length [8,9]. However, the importance of point light detection for reef fish is unclear.

### Hypotheses and data analysis

We tested for differences between nocturnal and diurnal eye morphology with several different techniques. All calculations were performed on the statistical platform 'R' (version 2.13.1) [39]. We calculated species means of the individual averages obtained from measurements of left and right eyes prior to all analyses. Then, we log10-transformed the data and rounded to four significant figures. Ratios were calculated directly from the original, untransformed species averages.

#### Differences between nocturnal and diurnal eye size

We tested whether nocturnal fish have larger eyes than diurnal species by Standardised Major Axis regression of eye diameter and body mass, performed with the R package smatr (as for all other regression analyses) [40]. We first calculated the slope for all species in order to understand the scaling of eye and body size among all reef fish. We then tested for differences in slope to determine if the slopes were equal. Finally, we compared intercepts between nocturnal and diurnal species. We chose body mass as the independent variable because it may better account for variability in body shape (e.g. long and slender versus deep-bodied and short) than the other commonly used size proxy, standard length. Anguilliforms, with their extremely elongated bodies and large mass but relatively small heads were not included in this part of the analysis.

#### Differences between nocturnal and diurnal eye morphology: Optical ratio and pupil shape

We empirically tested OPT with two sets of morphological proxies for optical variables. First we followed Schmitz and Motani's [27] approach and chose lens diameter (LD) as a proxy for optical aperture and eye diameter (ED) and axial length (AL) as proxies for retina diameter and focal length (note that focal length substitutes for PND in aquatic eyes):

$$\text{OP}{\text{T}}_{\text{morph1}}={\text{LD}}^{\wedge }2∕\text{(ED}\times \text{AL)}.$$

We then substituted lens diameter with the smallest pupil diameter PDmin, respectively) as another empirical test:

$$\text{OP}{\text{T}}_{\text{morph2}}={\text{PDmin}}^{\wedge }2∕\text{(ED}\times \text{AL)}.$$

We did not use the largest diameter of the pupil because this trait has two major functions. One function is related to optical aperture, while the other concerns lens accommodation. The elongation of the long axis of the pupil may result in an aphakic ("lensless") gap, which is considered useful for lens accommodation [41].

Lens diameter also serves two major optical functions in the teleost eye, which may compromise the distinction between nocturnal and diurnal reef fish. The lens ensures that most light entering the eye chamber is brought into focus by matching the size of the optical aperture, but is also the only refractive element in the teleost eye and determines the focal length. This means that, if everything else stays the same, any enlargement of lens diameter for a larger optical aperture may also result in a longer focal length, with f-number and possibly OPT remaining constant. Variation may be limited in particular concerning lens diameter and axial length, with the latter also being a proxy for focal length.

In order to test whether OPT_morph1 _and OPT_morph2 _are useful discriminators of diel activity patterns in teleost reef fish we plotted the numerators (LD^2; PDmin^2) against the common denominator (ED × AL). This approach avoids introducing size-dependent bias to the ratio by accounting for possible allometric scaling of involved variables. We fitted SMA regression lines to pooled data and also to nocturnal and diurnal species separately, comparing slopes and intercepts.

Furthermore, the pupil of nocturnal fish is expected to approximate a circular shape in order to maximize the area of the optical aperture. We tested this prediction by SMA regression of smallest and largest pupil diameter, where the regression line of nocturnal species should have a higher intercept than that of diurnal fish.

Finally we derived a new ratio, that combines aspects of OPT and geometry of the optical aperture. We modified OPT_morph1 _by cancelling out LD/AL because both traits are correlated with focal length and differences between nocturnal and diurnal groups may be limited, leaving LD/ED. Nocturnal species are expected to have larger LD (~optical aperture) for a given ED (~retina diameter) than diurnal species. Then, we combined LD/ED with the ratio describing pupil shape, PDmin/PDmax, where nocturnal species should have a large PDmin for a given PDmax, in order to maximize the pupil area. The combination of these ratios yields

$$\text{OP}{\text{T}}_{\text{morph3}}=\text{(LD}\times \text{PDmin)/(ED}\times \text{PDmax)}.$$

#### Differences between nocturnal and diurnal eye morphology: Multivariate analyses

We applied principal component analysis (PCA, correlation matrix) to further explore the eye-morphospace of nocturnal and diurnal reef teleosts. We performed two different PCAs. The first PCA included three variables, namely eye diameter, axial length, and lens diameter. These are the same variables used in the discriminant analysis in previous studies on terrestrial eyes [27,28,38]. The second PCA included these three variables plus the largest and smallest pupil diameter, in order to have a more complete description of eye morphology. For both PCAs we performed a MANOVA to test for differences between nocturnal and diurnal taxa.

Third, we tested whether linear, quadratic, regularized, or flexible discriminant analysis (LDA, QDA, RDA, and FDA, respectively) can successfully distinguish between nocturnal and diurnal eyes. Similar to the PCA, we first began with a set of three variables (eye diameter, axial length, and lens diameter), before adding in the largest and smallest pupil diameter as fourth and fifth variable. To determine the minimal misclassified proportion with RDA, we varied the regularization-lambda between 0 and 1 at increments of 0.01. LDA and QDA were performed with the R package MASS [42], FDA with the mda-package [43], and RDA with the klaR-package [44]. For all discriminant analyses we used prior probabilities defined by the training dataset.

#### Diversity of nocturnal and diurnal eye morphology

For the comparison of the diversity of nocturnal and diurnal eye morphology we analyzed the pattern of morphospace occupation defined by the PCA (PC axes 2-5) on all five variables. We assessed morphological diversity by means of variance. There are more diurnal (n = 211) then nocturnal species (n = 54) in our dataset, and even though variance is considered largely independent of sample size, we accounted for a possible bias by a rarefaction analysis. We randomly re-sampled 54 diurnal species without replacement and calculated variance on PCs 2-5, and repeated this procedure 100, 000 times. This procedure resulted in 100, 000 PC analyses with the same number of diurnal and nocturnal species, with diurnal species randomly selected anew for each run. Then, we compared the distribution of nocturnal variances to the bootstrap distribution of diurnal variances.

## Results

### Differences between nocturnal and diurnal eye size

**Figure 2:**
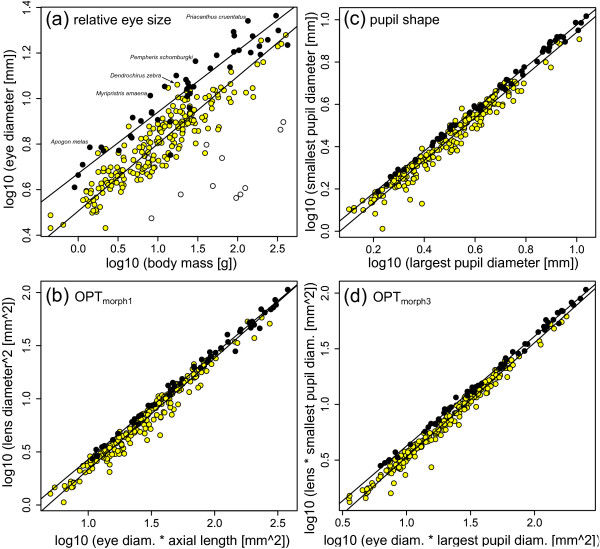
**Relative eye size and optical ratios of nocturnal and diurnal teleost reef fish**. Panel (a) shows that nocturnal reef teleosts (black circles) have larger eye diameter for given body mass than diurnal species (yellow circles). Open circles are elopomorphs, which have very elongated bodies that make a comparison with body mass as independent variable impossible. The plot of the square of lens diameter against the product of eye diameter and axial length (OPT_morph1_) reveals that nocturnal reef teleosts have a relatively large lens (b). Nocturnal fish have more circular pupils than diurnal fish (c), and if one combines the pupil ratio with OPT_morph1_, cancelling out the ratio of lens diameter and axial length, a fairly clear delineation between groups of diel activity pattern emerges (d).

We tested whether nocturnal reef fishes have larger eyes than diurnal species by regressing eye diameter against body mass with the Standardised Major Axis (SMA) technique. The resulting plot (Figure 2a) shows a distinct separation between nocturnal and diurnal species. Eye diameter scales with negative allometry against body mass in both nocturnal and diurnal subgroups. The slope of the regression line for nocturnal fish is 0.270 while the slope for diurnal fish is 0.296 and the probability of the slopes being equal to 0.333 (isometry) is smaller than 0.001. However, the slope of a regression fitted to both groups at once is 0.317, and the associated probability of the slope not being different from 0.333 (isometry) is 0.148. The slopes of nocturnal and diurnal species are not different (p = 0.126), which allows for meaningful comparison of intercepts: the regression line of nocturnal fish has a much higher intercept (0.671) than that of diurnal species (0.506; p < 0.001). The calculation of intercepts using the common slope (0.289) indicates that on average nocturnal reef teleosts have eye diameters about 1.355 times larger than the eye diameters of diurnal species for given body mass. The delineation between nocturnal and diurnal groups is most distinct for smaller fish, whereas there is considerable overlap among large fish.

### Differences between nocturnal and diurnal eye morphology: Optical ratio and pupil shape

We tested for differences in the optical ratio, the ratio of the square of optical aperture and the product of retinal diameter and posterior nodal distance (OPT, [27,28,38], please also see Methods) with several sets of morphological proxies. First, we plotted the square of lens diameter against the product of eye diameter and axial length (OPT_morph1_, Figure 2b). The diurnal and nocturnal groups have slightly, but significantly different SMA slopes (1.05 and 0.994, respectively; p = 0.003). Nocturnal fish tend to have larger squared lens diameters for a given product of eye diameter and axial length, but there is considerable overlap between groups especially for large eyes (Figure 2b).

The delineation between nocturnal and diurnal species with OPT_morph2 _is worse. Small nocturnal fish tend to have a slightly larger product of largest and smallest pupil diameter for given product of eye diameter and axial length, but the differences are absent for larger eyes. Again, the SMA slopes of the nocturnal (1.082) and diurnal groups (1.004) are different (p = 0.008).

The pupil shape of nocturnal and diurnal fish is different (Figure 2c). Nocturnal fish have a more circular pupil than diurnal fish, as the plot of the smallest against the largest pupil diameter shows. The slopes of the regression lines of nocturnal (0.994) and diurnal species (1.009) are similar (p = 0.371), but nocturnal fish have a much higher intercept (-0.021, compared to -0.063, p < 0.001). The circular shape of the pupils of nocturnal teleosts effectively reduces their aphakic gaps, i.e. the "lensless" part of the pupil. In diurnal fish, the lens diameter is 82.5% of the long pupil axis, which means that on average there is an aphakic gap equalling 17.5% of the largest pupil diameter. In nocturnal reef teleosts this distance is significantly smaller (10.3%, p < 0.001, t-test). Allometry does not affect this ratio, because lens diameter scales isometrically with the largest pupil diameter (p = 0.844).

The best delineation between groups of diel activity pattern is achieved by OPT_morph3 _(Figure 2d), even though some overlap remains. This ratio combines OPT with pupil shape, and excludes traits that are involved in functions other than light sensitivity (please also see Methods). Nocturnal species have a larger product of lens diameter and short axis of the pupil for a given product of eye diameter and long axis of the pupil. The slopes of the two regression lines are slightly different (nocturnal slope = 0.985, diurnal slope = 1.026, p = 0.001), with nocturnal species plotting above diurnal fish with comparably little overlap at least for small eyes. The differences become smaller for larger eyes (Figure 2d).

### Differences between nocturnal and diurnal eye morphology: Multivariate analyses

The Principal Component Analysis (PCA) performed on eye diameter, axial length, and lens diameter, the same variables used to calculate OPT_morph1_, also indicates that nocturnal species tend to have a larger lens for a given eye size than diurnal species. The first principal component (PC 1) explains most of the variance in the data (98.96%), and is characterized by nearly identical loading on its three components (eye diameter: -0.579, axial length: -0.576, and lens diameter: -0.577; Table 1). Scores on PC 1 are strongly correlated with the geometric mean of all three variables (p < 0.001, R-squared of SMA regression is > 0.999), in contrast to scores on PC 2 (p = 0.975, R-squared < 0.001) and PC 3 (p = 0.504, R-squared < 0.001). Hence, PC 2 and 3 represent differences in shape alone. PC 2 is formed by positive loading on lens diameter (0.64) and also eye diameter (0.118), but negative loading on axial length (-0.759), in contrast PC 3 is formed by positive loading on eye diameter (0.807) and negative loading on both lens diameter (-0.507) and axial length (-0.303). On the basis of these loadings species with high optical ratio, i.e., the ratio of the square of lens diameter and the product of eye diameter and axial length should plot in the bottom right quadrant of morphospace.

**Table 1:** Loadings on principal component axes

*PCA with three variables*		loading on principal component axes				
	abbreviation	PC 1	PC 2	PC 3	PC 4	PC 5
eye diameter	ED	-0.579	0.188	0.807	-	-
axial length	AL	-0.576	-0.759	-0.303	-	-
lens diameter	LD	-0.577	0.640	-0.507	-	-
percentage of variance explained		98.96	0.74	0.29	-	-
*PCA with five variables*		loading on principal component axes				
	abbreviation	**PC 1**	**PC 2**	**PC 3**	**PC 4**	**PC 5**
eye diameter	**ED**	-0.448	0.371	0.169	-0.424	0.673
axial length	**AL**	-0.446	0.586	-0.368	0.536	-0.190
lens diameter	**LD**	-0.449	0	0.519	-0.311	-0.655
max. pupil diameter	**PDmax**	-0.446	-0.467	-0.67	-0.354	0
min. pupil diameter	**PDmin**	-0.446	-0.546	0.345	0.558	0.268
percentage of variance explained		98.03	1.1	0.4	0.31	0.15

**Figure 3:**
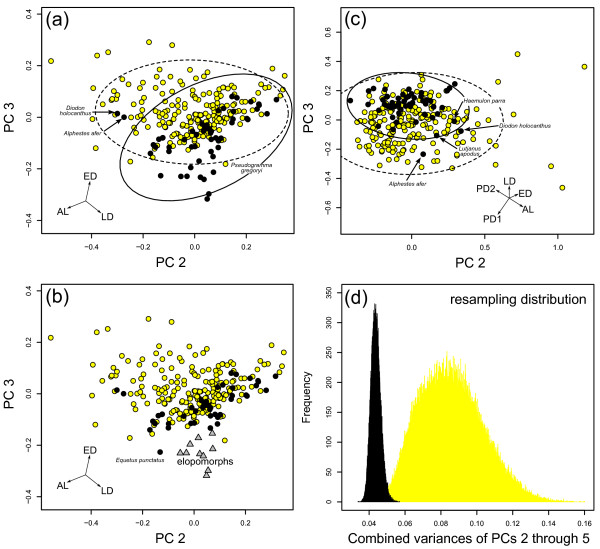
**Principal component analyses (PCA) of eye measurements**. We performed two different principal component analyses. One PCA was carried out using three variables (eye diameter, axial length, and lens diameter) and the resulting scatterplot of scores on PC 2 and PC 3 is illustrated in (a) and (b)). Panel (a) shows 95% confidence ellipses for nocturnal and diurnal groups; panel (b) demonstrates that elopomorphs occupy distinct areas in morphospace. The second PCA was carried out with five variables (adding largest and smallest pupil diameter; (c)). Vectors indicate magnitude and direction of loadings on PC axes. The plot of PC 3 against PC 2 shows in both analyses that nocturnal teleost reef fish (black circles) occupy different areas in morphospace than diurnal reef teleosts (yellow circles). Nocturnal species occupy a smaller area of morphospace. This is supported by the low combined variance of PC scores on all PC axes, calculated for the PCA with five variables, the most complete description of eye morphology available. Uneven sample size does not drive this pattern as shown by the resampling distribution for diurnal (yellow) and nocturnal (black) species. Ellipses represent 95% confidence ellipses for nocturnal and diurnal groups.

Indeed, nocturnal species in our dataset tend to occupy the lower right corner of the plot of PC 2 and PC 3 (Figure 3a), even though there is considerable overlap with diurnal fish. Nocturnal species that are found in areas in morphospace outside the diurnal area (minimum polygon fit) include most elopomorphs (Figure 3b), and the sciaenid *Equetus punctatus*. The nocturnal diodontid *Diodon holocanthus *and the serranid *Alphester afer *are deep within the diurnal morphospace, whereas the diurnal serranid *Pseudogramma gregoryi *is far in the nocturnal morphospace. Despite the overlap a MANOVA on the scores of PC 2 and 3 suggests differences in average shape between nocturnal and diurnal groups (p < 0.001).

The PCA performed on the complete set of eye variables (eye diameter, axial length, lens diameter, largest and smallest pupil diameters) reinforces differences between nocturnal and diurnal reef fish. Again, PC 1 contains most of the variance (98.03%) and is strongly positively correlated with the geometric mean of eye variables (p < 0.001). All variables load uniformly on PC 1, ranging from -0.449 to -0.446. PC 2 through 5 are independent of size and reflect differences in eye shape (PC 2, p = 0.963; PC 3, p = 0.877; PC 4, p = 0.431; PC 5, p = 0.701; R-squared for all slopes (SMA regression) < 0.001). Both largest and smallest pupil diameters load negatively on PC 2 (-0.467 and -0.546), whereas eye diameter and axial length load positively (0.371 and 0.586). Lens diameter has no effect on PC 2. PC 3 is formed by positive loading on eye diameter (0.169), lens diameter (0.519), and smallest pupil diameter (0.345), while axial length (-0.368) and largest pupil diameter (-0.670) have negative loading on PC 3. For a full list of loadings on all PC axes please refer to Table 1.

**Figure 4:**
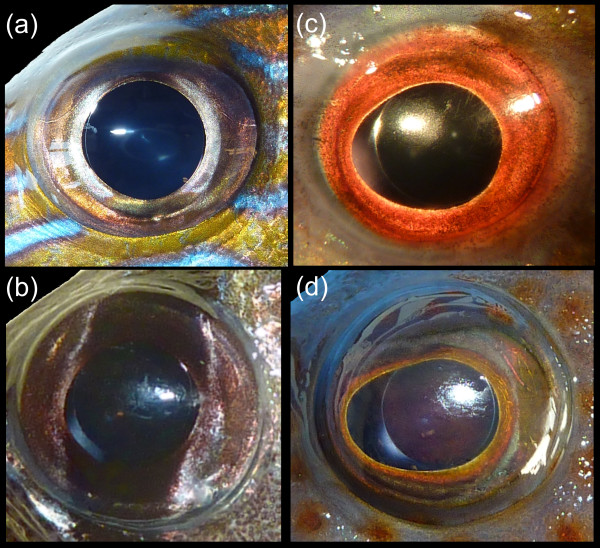
**Eyes of nocturnal and diurnal teleost reef fishes**. Lateral view of the left eyes of nocturnal ((a) the haemulid *Haemulon sciurus*, (b) the apogonid *Pterapogon kauderni*) and diurnal ((c) the labrid *Clepticus parrae*, (d) the serranid *Epinephelus cruentatus*) reef teleosts. Note the large rounded pupil in nocturnal fish, and the pronounced rostral aphakic gap in *Epinephelus cruentatus*. Not to scale.

The plot of PC 3 versus PC 2 (Figure 3c) reveals that nocturnal reef fish are mostly confined to the top left quadrant of eye morphospace, i.e., nocturnal species have low scores on PC 2 and high scores on PC 3. On the basis of the loadings on PC axes, low scores on PC 2 indicate large pupil size for a given eye size. High scores on PC 3 indicate round pupil shape (maximizing smallest pupil diameter for given largest pupil diameter) and a large lens for a given axial length and largest pupil diameter. Hence, nocturnal reef teleosts have relatively large and rounded pupils, and a large lens (Figures 4a, b), whereas diurnal teleosts tend to have smaller, more elliptical pupils (Figures 4c, d). There is no strict delineation in morphospace between groups; diurnal reef fish widely overlap with nocturnal species. Again, the nocturnal diodontid *Diodon holocanthus *and the serranid *Alphester afer*, this time along with the lutjanid *Lutjanus apodus *and the haemulid *Haemulon parra*, are deep within the diurnal morphospace. However, a MANOVA on the scores of PC 2 and 3 suggests a significant difference in average shape between nocturnal and diurnal species (p < 0.001).

Discriminant analysis (DA) of eye variables confirms differences between nocturnal and diurnal groups among reef fish. DA of eye diameter, axial length, and lens diameter - equivalent to the approach of Schmitz and Motani (27, 38) - yields a misclassified proportion of 12.45% for linear and flexible DA and 11.7% for quadratic DA. Regularized DA performed slightly better with 10.94% misclassified at regularization-lambdas of 0.55-0.74.

The inclusion of two additional variables (largest and smallest pupil diameter) slightly improves the discrimination. The misclassified proportion for linear, quadratic, and flexible DA is 10.19%. The lowest misclassification results (8.3%) are with a regularized DA, at regularization-lambdas of 0.1-0.11 and 0.14-0.18.

There are several nocturnal species that are classified as diurnal by all applied DA types, using either three or five variables. In particular apogonids have a large number of consistently misclassified species (*Apogon margaritophorus*, *Apogon townsendi*, *Sphaeramia nematoptera*, *Zoramia leptacantha*). Other consistently misclassified species are the tetraodontiform *Diodon holocanthus *and the serranid *Alphestes afer*, both of which are clear outliers (Figures 3a, c), the haemulid *Diagramma picta*, as well as the holocentrid *Myripristis amaena*. Similarly, all applied DA types classify two diurnal species as nocturnal: the mullet *Mugil cephalus *and the lionfish *Dendrochirus zebra*. The lionfish also has very large relative eye size (Figure 2a).

### Diversity of nocturnal and diurnal eye morphology

We assessed the diversity of eye morphology by means of the morphospace defined by all shape axes (PC axes 2-5) of the PCA of all five eye variables. The variance of diurnal species (0.105) is about twice as large as the variance of nocturnal species (0.055). The rarefaction test (re-sampling without replacement) that accounts for uneven sample sizes in diurnal (n = 211) and nocturnal sub-groups (n = 54) suggests that nocturnal species indeed have lower variance in eye morphology than diurnal fish (p < 0.001) (Figure 3d). The bootstrap distributions of nocturnal and diurnal species have two clearly separate peaks. Similarly, nocturnal species have much lower variance in all morphological approximations of OPT (OPT_morph1_, nocturnal variance = 0.0005: diurnal variance = 0.001; OPT_morph2_, 0.0015:0.0031, OPT_morph3_, 0.0006:0.0013).

## Discussion

Light levels are considered a major physical factor in the evolution of eye shape [29,45-47]. Diel activity patterns are the main mechanism controlling ambient light levels of teleost reef fish. If light levels do indeed impact the evolution of eye morphology in reef fish, then nocturnal and diurnal species are expected to differ in eye size and shape. We also postulated that stabilizing selection for traits that improve light-sensitivity limits morphological and functional diversity in nocturnal species. Our results confirm all hypotheses, even though shape differences are more subtle than what is observed in terrestrial amniotes [27], which may be due to constraints of aquatic vision. The observed patterns for relative eye size and optical ratios indicate that differences between nocturnal and diurnal eye morphology become less pronounced for absolutely larger eyes. This may indicate that neural summation by pooling of photoreceptor signals becomes more important in larger eyes, where the negative effects of decreased acuity by summation could be at least partially mitigated by the increase of focal length with eye size.

The optical system of diurnal reef species, which are active in mostly bright light environments, is characterized by photopic image formation. Some diurnal fish on reefs may experience lower light levels depending on microhabitats that locally reduce light availability, such as crevices, reef overhangs, or other three-dimensionally complex reef structures. Light levels exponentially decrease with water depth, yet light levels equivalent to star-lit night conditions on land are not reached until a depth of approximately 600-700 m in clear ocean waters that characterize most coral reef environments [48]. As far as currently known, none of the species in our dataset are active at such depths. Even if one assumes coastal visibility conditions for reefs, diurnal species do not experience the dim ambient light levels of their nocturnal counterparts during their diel activity period. Several species in our dataset are known to enter water with large amounts of suspended sediment and lower ambient light levels. One of these species, the mullet *Mugil cephalus*, appears to show characteristics of scotopic vision, but all in all it is diel activity pattern that defines the ambient light levels experienced by the species we sampled. Nocturnal fish need to rely on scotopic image formation and there is a clear perception of how the optical system of nocturnal species should be shaped in order to meet the requirements of scotopic vision [27].

### Differences between nocturnal and diurnal eye size and morphology

Our results confirm the predictions from physiological optics. Nocturnal teleost reef fish have much larger eyes for given body mass than diurnal species, as shown by the regression of eye diameter on body mass (Figure 1a). On average, eye diameter of nocturnal species is about 1.4 times larger than that of diurnal reef teleosts for given body mass. We assessed differences in eye shape by means of morphological proxies of the optical ratio (Figures 2b-d) and also multivariate analyses (PCA, Figures 3a-c, and DA). The eyes of nocturnal reef teleosts differ from their diurnal counterparts in that they have relatively large lens diameters and large, rounded pupils. These characteristics should increase the amount of light transmitted compared to a smaller lens and smaller or asymmetric pupils. More light transmission should enhance the brightness of the retinal image, which will translate into better scotopic vision for a given retinal structure and neurological processing. Interestingly, the differences in relative eye size and eye shape fade to some degree for larger species. The negative allometry observed in the scaling of eye diameter with body mass is in the range previously reported for fish [18,22,49], although elasmobranchs seem to have much stronger negative allometry [50].

The characteristics of a nocturnal eye seem to be present in nocturnal species independent of phylogeny, indicating convergent morphological evolution. We sampled nocturnal species from 12 different families across Elopomorpha and the acanthomorph clades Holocentridae and Percomorpha, and most species show nocturnal characteristics. It is not clear at this point how many independent nocturnal lineages are included in our dataset, because the phylogenetic relationships of these clades are not well studied. In particular the phylogenetic relationships among families of acanthomorphs, a group containing more than 16, 000 extant species, is currently poorly understood [51] and is one of the most challenging problems in vertebrate phylogenetics. On the basis of the current understanding of teleost phylogeny [51-53] and a conservative approach, there are at least seven independent origins of nocturnality represented in the data. There is at least one origin of nocturnality within elopomorphs (Congridae, Muraenidae, Ophichthidae) and one within holocentrids. Within percomorphs, there are probably more than five independent origins: apogonids+pempherids (Apogonoidei) as possible sister group to the diurnal Gobioidei [54], one each within the largely diurnal Serranidae (e.g., *Alphestes*) and Tetraodontiformes (e.g., *Diodon*), and all other sampled nocturnal families, i.e., Priacanthidae, Haemulidae, Sciaenidae, Lutjanidae, which conservatively, even though unlikely, may represent a single nocturnal radiation.

The apparently constraining requirements on eye shape in nocturnal teleost reef fish are similar to what has been observed in terrestrial amniotes, where the strongest correlation between structure (OPT) and function (ocular image formation) is found at Pagel's λ of 0.01 [28]. Pagel's λ is a measure of phylogenetic signal in the data, and a value of nearly zero indicates minimal phylogenetic signal [55]. It is possible that the phylogenetic bias in eye morphology of teleosts is somewhat larger, yet this cannot be evaluated until a reasonably well-resolved and time-calibrated phylogeny at the species level is available.

Phylogenetic bias may be part of the reason why the differences between eye shape of nocturnal and diurnal teleost reef fish appear not as distinct as in terrestrial amniotes. For example, a comparison of DA results for the same measurements in each dataset (eye diameter, axial length, lens diameter) shows that the misclassified proportion tends to be larger within reef teleosts (10.94%) than in terrestrial amniotes (4.92%, regularized DA, spherical eyes, [27]). A phylogenetically-informed DA [28] can potentially improve correct classification.

It is also necessary to consider differences between vision in air and in water. The cornea does not function as a refractive surface in water, leaving the lens to provide all the light refraction required to focus the image in an aquatic eye [56-58]. Thus, the lens alone performs two main functions of the optical system. First, the lens focuses light onto the retina (assuming emmetropia) and sets the focal length. In terrestrial eyes both cornea and lens provide this function, and there is some variation in the proportional contributions to the total refractive power of the eye [59] that leaves opportunity for morphological diversity. This variation is absent in aquatic eyes, which are more or less built according to Matthiessen's ratio [58]. In order to keep the eye and focal length at a reasonable size, the refractive power of the lens needs to be strong [16,58]. The increase in refractive power can be realized by shortening the radius of curvature, resulting in a nearly spherical, small lens [58-60]. Second, lens diameter is correlated with pupil diameter, i.e., the aperture of the optical system, ensuring that most incoming light is focused onto the retina and does not cause any blur and scattering. The dual function may be an inherent structural limitation that renders further improvement of scotopic image formation difficult. For example, if an eye had a larger lens for a larger aperture, the larger radius of curvature of the lens would also increase focal length, provided everything else stayed the same. However, there is some indication that the eyes of nocturnal reef fish have shorter focal lengths [61], which may help to at least partially overcome the structural limitation of having only one refractive element. More data are needed to better understand potential differences between optical qualities of the lens of nocturnal and diurnal species.

Further research is also needed for an improved understanding of diel activity patterns in reef teleosts. There is a pronounced nocturnal-diurnal turnover at dusk and dawn among reef fish [5,62,63], but it is likely that a dichotomous split into nocturnal and diurnal species does not fully capture the complexity of temporal resource and habitat partitioning. Indeed, there is evidence that some reef teleosts may be active day and night, but current data are insufficient to have a clear understanding of possible cathemeral (day- and night-active) or crepuscular (twilight-active) behaviour. Furthermore, some species may display plasticity in their diel activity pattern [34]. A third category as used in the analysis of terrestrial amniotes [27,28] may further improve the delineation between groups. Some of the nocturnal and diurnal species that overlap in discriminant space may in fact be cathemeral or crepuscular species, which are expected to be intermediate in shape and light-gathering power. Clearly, more behavioural data are needed to further investigate diel activity patterns in reef fish.

The eye shape of some nocturnal species does not match the evolutionary response to scotopic vision seen in other reef fish, even though their nocturnal behaviour generally seems to be well supported. The pufferfish *Diodon holocanthus *is nocturnal as an adult, but settles from a planktonic mode of life at a standard length of 100-120 mm [personal communication, D.A. Bellwood]. As the three specimens of *D. holocanthus *that we sampled are all around 105 mm, we cannot fully exclude that there is an ontogenetic effect biasing the characterization of the ocular morphology of this species. In addition, some nocturnal species with ocular morphology not matched to scotopic vision may rely on non-visual senses (e.g., olfaction, lateral line system). Alternatively, they may solve the problem of vision at low light levels by modifications of parts of the visual system other than ocular morphology, e.g., at the level of the retina. It will be important in the future to expand this study to include additional features related to optical sensitivity, for example the diameter and length of photoreceptors [9,37].

### Morphological and functional diversity of nocturnal and diurnal eyes

One of the main objectives of this paper was to characterize the pattern of eye morphospace occupation. We chose the morphospace of the PCA performed on all five measured variables (PC 2-5), representing the most complete representation of eye shape. The results show convincingly that nocturnal species are restricted to a small area of morphospace compared to diurnal species. Functionally, the area of nocturnal species is related to improved scotopic image formation. Diurnal species, which are released from the physical limitations of scotopic vision, have a much wider morphospace occupation. Diurnal fish occur within the area of good scotopic vision, but also explore other parts of morphospace such as areas that are characterized by small pupil area for given eye size. Intriguingly, there is a trade-off between scotopic image formation and depth of focus, because a large pupil is positively correlated with light sensitivity yet negatively linked with depth of focus [60]. In addition, some diurnal species have strongly elliptical pupils with often rostrally placed aphakic gaps, which supposedly enable them to focus on close objects in the anterior field of view, and may also allow for binocular vision [41]. An ability to focus on close objects in the anterior field of view may be helpful to select benthic prey items. The presence of other adaptive peaks, in combination with the release from physical limitations of scotopic image formation results in a larger morphospace occupation of diurnal species compared to that of nocturnal species.

It is unlikely that this pattern of morphospace occupation is strongly influenced by phylogenetic bias. There are two possible phylogenetic mechanisms that would result in low variance in nocturnal taxa. First, if all nocturnal species were from a monophyletic clade they would be expected to be similar morphologically simply because of their shared evolutionary history. As explained earlier, we sampled at least seven independent origins of nocturnality, which should reduce this possible phylogenetic bias. Second, if all nocturnal radiations are very young compared to diurnal clades and one assumes a Brownian model of evolution, then the nocturnal clades are expected to have low variance, simply because they had less time to diversify [64,65]. Time-calibrated phylogenies are not available yet and it is difficult to estimate the basal node ages of nocturnal and diurnal clades. However, the fossil record indicates that most nocturnal groups are of approximately the same age as diurnal groups, as many extant reef teleost families appear in the Eocene [19,66]. The appearance of a large number of clades in a geologically brief time interval is congruent with the difficulty to resolve phylogenetic relationships of the percomorph "bush". We also attempted to sample widely within a given clade, both in terms of geographic provinces and known phylogenetic relationships, in order to avoid sampling a geologically young sub-clade. All in all, the phylogenetic influence on morphospace occupation should be small, and the low variance seen in the morphology of nocturnal reef fish seems to be the result of stringent functional requirements.

It will be an interesting avenue of future research to analyze how the eyes of mesopelagic (150-1,000 mm depth) fish have met the functional requirements of scotopic vision. For example, the presence of circumlental aphakic gaps in some deep-water species has been interpreted as a mechanism to improve sensitivity to point light sources like bioluminescent flashes [67,68]. Warrant and Lockett [69] have suggested that there are two main eye shapes among these deep-water fish: the familiar ellipsoidal eye shape of shallow-water reef fish, and a tubular eye shape reminiscent of the eyes of owls and nocturnal primates, with large, spherical lenses, large pupils but small eye diameter for given axial length. Tubular eyes apparently represent a different solution to the problem of scotopic vision. Our data suggest that nocturnal reef teleosts have not followed these evolutionary pathways.

## Conclusions

The requirements of scotopic image formation of nocturnal reef teleosts are met with a series of modifications of eye morphology that are considered to improve light sensitivity. These modifications come at the cost of a smaller depth of focus and strong reduction of aphakic gaps. Diurnal species, freed from physical limitations of scotopic vision, have higher morphological and optical diversity.

Diurnal fish also display enormous trophic diversity. All major feeding guilds, i.e., herbivores, carnivores, and omnivores are represented in the diurnal group, including specialists feeding on mobile and sessile benthic prey, coral mucous, algae, nekton, zooplankton, and ectoparasites [1,4,70]. This variety contrasts the limited trophic diversity of nocturnal fish, which are mainly carnivores. Common prey items of nocturnal fish are restricted to large elusive prey, mobile benthic invertebrates, and large zooplankton [1,2,13,15]. This uneven distribution of trophic diversity in nocturnal and diurnal teleost reef fish may possibly be related to the physical challenge of prey detection at night and modalities of sensory systems. In particular the visual system is important for understanding ecological radiations in nocturnal and diurnal lineages, because most reef fish are highly visual foragers [10,11].

To conclude, diel activity patterns emerge as a major factor in the morphological evolution of the eyes of both terrestrial amniotes and teleost reef fish. In both clades the physical requirements of scotopic vision are met with similar morphological and optical modifications, highlighting the important role of physical characteristics of the environment in the evolution of morphology.

## Authors' contributions

PCW and LS developed ideas and designed the study. PCW collected specimens while LS collected data, performed analyses and wrote the manuscript, with final edits made by PCW. All authors read and approved the final manuscript.

## Supplementary Material

Additional file 1 **Eye morphometrics and diel activity pattern of teleost reef fish**. Click here for file
